# Artificial Pancreas: A Review of Meal Detection and Carbohydrates Counting Techniques

**DOI:** 10.1900/RDS.2022.18.171

**Published:** 2022-12-31

**Authors:** Edward Rodriguez, Rodolfo Villamizar

**Affiliations:** Universidad Industrial de Santander, from Bucaramanga, Colombia.

**Keywords:** diabetes, artificial pancreas, meal detection, meal estimation, closed loop

## Abstract

**OBJECTIVE:**

The development of an artificial pancreas is an open research problem that faces the challenge of creating a control algorithm capable of dosing insulin automatically and driving blood glucose to healthy levels. Many of these approaches, including artificial intelligence, are based on techniques that could result in and undesirable outcome because most of them include neither detect meal intake or meal size information. To overcome that issue, some meal count-detection algorithms reported in scientific publications have shown not only a good performance on blood glucose regulation but fewer hypoglicemia and hyperglycemia events too.

**METHODS:**

We reviewed the most relevant authors and publications and main databases (particularly SCOPUS and Google Scholar), focusing on algorithms of detection and estimation of meal intake from multiple approaches.

**RESULTS:**

A wide range of approaches and proposals have been found. The majority of them include trials on in silico patients rather than in vivo ones. Most of procedures require as inputs glucose samples from continuous glucose monitoring devices as basal insulin and bolus as well. Most of approaches could be grouped by 2 categories: mathematical model based and not model based.

**CONCLUSION:**

A combination of methods seems to reach better results.

## 1 Introduction

According to the World Health Organization [1] type I diabetes mellitus (T1DM) is a chronic disease that occurs either when the pancreas does not produce enough insulin or when the body cannot effectively use the insulin it produces. According to the International Diabetes Federation (IDF) [2], there are about 463 million people worldwide with diabetes and it is estimated that by 2045 there will be 700 million people with this disease. The IDF also estimates that between 2019 and 2045, diabetes will increase by 55% in Central and South America, becoming the fourth highest rate in the world. The national prevalence of diabetes in Latin America varies markedly across countries, with Peru registering the lowest (6.6%) and Puerto Rico the highest (13.7%). In addition, if diabetes’ treatment is inadequate, patients can experience undesired events such as hyperglycemia or hypoglycemia due to high or low blood glucose concentrations. Furthermore, poorly controlled diabetes can lead to complications, not only in the pancreas, but also other organs of body such as the kidney (kidney failure), leg (leg amputation), eyes (vision loss, glaucoma, diabetic retinopathy) and vascular problems. Some approaches have been proposed that aim for controlling a high blood glucose concentration through intensive insulin therapy (IIT) [3]. IIT is a set of strategies intended to imitate the same behavior patterns as insulin secretion in healthy subjects. In IIT, 2 common techniques are employed – multiple daily injections (MDIs) and continuous subcutaneous insulin infusion (CSII). However, these techniques require human intervention and are not always able [4] to reduce hypoglycemic episodes. Consequently, technology is moving towards development of an automatic delivery glucose system – the artificial pancreas (AP).

The AP, first proposed by A.H. Kadish [5], is today the best possible solution for insulin treatment, especially for type I diabetes patients [6]. It consists of a continuous glucose monitoring system (CGM), which consists of a glucose measurement device, a digital controller (computes insulin dose to be released), and finally, an insulin pump. Moreover, although the AP is designed to work without human intervention and ensure a good performance by reducing hyperglycemic episodes, it still faces a challenging task when meal intake is undetected, finding it workable only with (detected) small meal intake sizes [7].

The estimation of carbohydrate intake plays an essential role in the development of control systems focused on the regulation of glucose levels in diabetic patients. Different proposed control techniques [8–10] suggest the need for a carbohydrate intake estima- tor with targets that stand out – generating variable reference glucose profiles to avoid aggressive control efforts, calculating insulin boluses [11], and developing algorithms that allow this calculation to be carried out automatically for safety reasons in patients [12]. Regardless of the purpose, they converge on the same idea, i.e., a correct estimate of carbohydrate intake that leads to avoiding undesirable situations such as hypoglycemia and hyperglycemia, guaranteeing a greater sense of well-being in diabetic patients. In fact, they have been proposed and are usually included for 2 main reasons: estimation of intake based and not based on the model. The first proposes the use of a model whose parameters must be tuned, for example, using an MHE algorithm and a second-order system for glucose-insulin dynamics [13], or by using a combined unscented Kalman filter. with Bergman’s minimal model. This leads to an identification of parameters for each patient, which does not guarantee generalized use of the model. The second, raises the possibility of making this estimate by means of techniques in which a representation is not required. Mathematics that models glucose-insulin dynamics in patient highlights fuzzy logic algorithms [14] or carbohydrate estimation through image pattern recognition [15]. The rest of the paper is organized as follows. Section 2 describes the most frequently used approaches for dealing with meal detection and estimation. Section 3 covers the most commonly used datasets where the algorithms in section 2 have been tested. Finally, a brief discussion and results is presented in section 4.

## 2 Meal detection and estimation techniques

In this section, the most common techniques of meal detection and size estimation are addressed are the fundamentals and principal outcomes.

### 2.1 Heuristic Approaches

We label as heuristic those non-exact approaches that could work based on available information or prior knowledge and that are not intended to ensure an optimal solution.

#### 2.1.1 Decision rules

Decision rules are often related to a 2-step procedure. First, a set of relevant signals in the context of the problem is chosen (pre-processed if required). Second, the selected features are brought into a decision logic rules system (comparisons or thresholds around feature’s values). Finally, an output is provided. Decision rule systems are based on thresholds of glucose derivatives (glucose rate of change), the area under the curve (AUC), to announce when a meal is detected. One of most cited related works is called the voting-based detection system [58].

**Figure 1.**
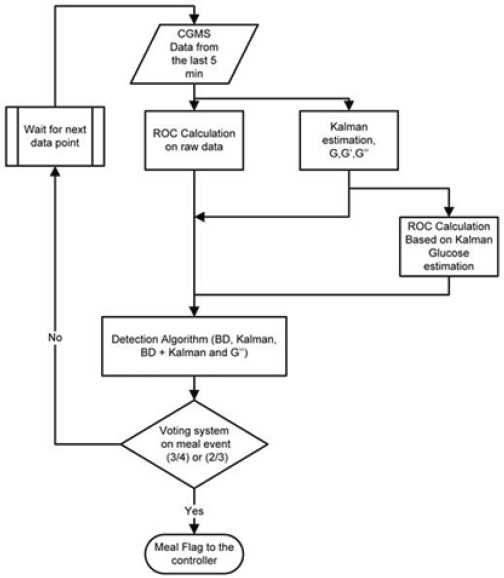
Meal detection algorithm voting scheme [58].

The scheme depicted in Figure 1 consists of computation of rate of change (ROC) in different ways, using backward difference on raw data, backward difference estimation based on the glucose estimation from the Kalman filter, Kalman filter estimation of glucose (G) and the ROC (G) (Kalman), and finally, the Kalman estimate of the ROC of the ROC (G). They are then compared with threshold values tuned according to individual patients. They are: glucose ROC threshold, maximum glucose ROC, glucose threshold, and an acceleration threshold. Similar approaches exist [59,60].

#### 2.1.2 Fuzzy Logic

Fuzzy logic is a slighty more complex approach than simple previous threshold-based decision logic. Here, a set of inputs is transformed into fuzzy sets. Fuzzy sets assign a degree (membership degree functions) for each input within a category according to a user-specified threshold. Then, different rules (mathematically seen like operations such as sums or products between different fuzzy sets) can be designed and a weighted output can be generated from the rules.

Analogously [14], glucose and its derivatives are used for a fuzzy inference system from which derivatives are computed by numerical differentiation. Then, to feed each first and second derivatives to the fuzzy system they are mapped by a membership function, each one with fuzzy sets: positive, negative, and zero. This is not more than weighing the positive/ negative/zero increasing of derivatives. Once the mapping is done, 7 in- ference rules (called episodes by the author) are established and weighted; finally, some auxiliary variables are computed and by using a decision rules systems, brings flags for meal detection and carbohydrate counting. Other steps and auxiliary variables are also required; hence, we strongly encourage the reader to follow the original paper for better understanding.

### 2.2 Machine Learning Approaches

**Figure 2.**
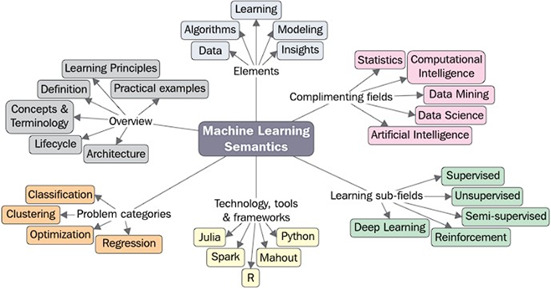
Key aspects in machine learning. Taken from [50].

Machine learning is a mixture of statistics, mathematics, programming, and optimiza-tion knowledge, often with purpose of getting better understanding of the relationtionships in a set of data. It is a branch of artifical intelligence and can be applied to fields like industry, medicine, research, stocks, sales, and computer vision [45], focused principally on prediction and classification tasks [Figure 2].

#### 2.2.1 Recurrent Neural Networks

**Figure 3.**
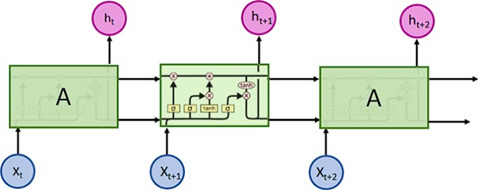
Intuitive recurrent neural network ilustration [29].

In neural networks exist some relevant architectures are particularly relevant: feed-forward, recurrent, convolutional, generative adversarial, and transformer neural networks. However, because recurrent neural networks have been used more for diabetes prediction and detection rather than the other architectures, this paper only covers that approach. Recurrent neural networks, also called RNNs, are a type of artificial neural network that adds additional weights to the network to create cycles in the network graph to maintain an internal state [28]. That network is supposed to establish long-short term patterns or dependencies between (usually time-dependent) data [Figure 3].

**Figure 4.**
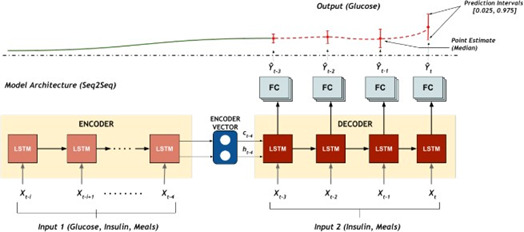
Multitask deep neural network architecture for predicting glucose [49].

As RNNs weigh the order of the data (or sequence), they keep them useful for some interesting tasks like natural language processing, or forecasting [30– 36]. Long-Short Term Memory (LSTM) networks are a subtype of recurrent neural network; they are more strongly used than traditional RNNs because of their capability of dealing with the vanishing gradient problem [37]. Therefore, in this field, it is possible to find RNN approaches by using LSTMs and ensemble learning strategies the model receives as input sequences of continuous glucose monitoring measurements. They are then classified as 2 categories, true or false [38]. One author [49] proposes an algorithm based on a deep learning for multitask quantile regression by using a sequence-sequence (seq2seq) encoder- decoder LSTM for predicting the last 20 minutes of glucose using historical CGM measures, meals, and insulin [Figure 4].

The encoder LSTM input comprises the glucose concentration levels from the CGM, insulin delivered and meals, and returns the encoder state representations. The decoder generates the output sequence and estimated quantiles of the glucose trajectory are generated at the output. The decoder input comprises the insulin delivered and announced/ estimated Meals, and is initialized with the final encoder state. The output sequence generated from the decoder is then fed to the 3 final layers of the model that consist of 3 separate tasks. Each task represents a quantile distribution, and consequently, the model performs a quantile regression for the associated quantile. The meal estimation and counting results in a flag detection and an iterative meal search respectively. As the LSTM outputs multiple quantiles for a 95% prediction interval, any prediction outside this interval supposes an unannounced input like a meal. The meal counting determines then, in an iterative search for meal value, which is the better fit for glucose trajectory.

#### 2.2.2 Binary Classification Algorithms

A common algorithm for classification is random forest, this is an ensemble learning method [40] for either classification or regression and it works by combining decision trees [41]. For classification tasks, the output of the random forest is the class selected by most trees. In diabetes, we can find a procedure where random forest and boosted tree models allow us to determine how some features like glucose, heart rate, physical activity, core temperature, skin temperature, and respiration rate may be utilized to develop an automated tool for meal detection in (metabolically) healthy participants [42]. A similar approach is found which uses an isolated trees algorithm instead [23]. Other binary classification techniques [24] include naive bayes and logistic regression being probably used for data mining [51– 53].

#### 2.2.3 Reinforcement learning

**Figure 5.**
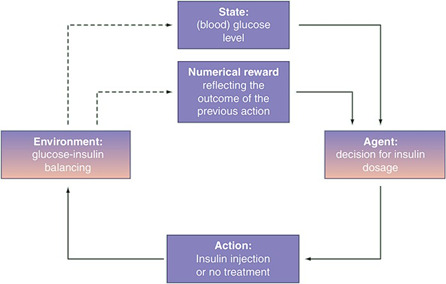
A basic scheme of reinforcement learning applied to the artificial pancreas problem [22].

This field is about the process of learning what to do-how to map situations to actions- so as to maximize a numerical reward signal [20] [Figure 5].

A reinforcement learning (RL) approach can be found for treatment of diabetes [21]. The author proposes a bioinspired RL designed for automated insulin infusion. The method includes reward functions that imply the temporal homeostatic objective and discount factors that reflect individual specific pharmacological characteristics. As in the previous example, it is possible to find similar methods or trials for controlling glucose; however, most of them are unable to estimate or to detect meal intake directly [22].

### 2.3 Control systems theory

Control systems theory is about measuring and controlling signals or variables com- mon to a physical process. The variable is modeled by time domain equations (differential equations) or frequency domain (transfer functions).

#### 2.3.1 Disturbance observer based control

**Figure 6.**
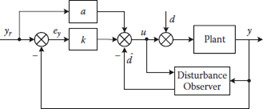
A basic scheme of disturbance obser ver based control [17].

As the process variable is supposed to be controlled, there also exist others undesirable signals that should be avoided because they can adversely affect the controller; they are also known as disturbances. Fortunately, there also exist some control techniques that aim to overcome disturbance effects – perhaps the most important of which is the disturbance observer based control (DOBC) [Figure 6].

**Figure 7.**
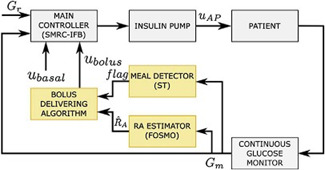
Proposed scheme [18].

In the context of diabetes, meal intake can be seen as a main disturbance because this drives glucose levels into hyperglycemia events and prevents reaching normal glycemic levels; in that sense, DOBC looks for estimates and to reject meal intake. In one study [16], a DOB-based control is proposed to deal with undetected meals. The observer estimates unexpected variations in the glucose level by using the information of the selected features: glucose (from a continuous glucose monitor), insulin infusion rate, and a subject-dependent model. Another inner approach inside DOBC theory is about using sliding mode control (SMC). SMC has fine abilities in suppressing effects of parameter deviations as well as exter- nal disturbances [17]. In a related approach [18], the author exploits the advantages of sliding mode observers (SMO) due to their inherent robust properties. Furthermore, SMO can reconstruct disturbances. The author proposes an estimate of the rate aparison of glucose (RA) which is obtained via a first-order sliding mode observer (FOSMO). On the other hand, a metal detector algorithm is proposed based on a super- twisting observer (ST) to detect the faults (meals). More information about the super-twisting observer is available [19] [Figure 7].

#### 2.3.2 Model predictive control

As DOBC, the model predictive control (MPC) is nothing other than an alternative to the conventional techniques for controlling variables in a process. MPC was originallyintended to ensure optimal performance to compute appropiate control signals as dealing with constraint as well. More details are available in the literature [26]. Examples of MPC for diabetes also are reported in the literature [25]. For this approach the author presents a control system that automatically delivers priming boluses and/or anticipates eating behaviors to improve post-prandial full closed-loop control. The anticipating is achieved by detecting large glycemic disturbances. Additionally, to deliver an appropiate insulin dosage a group of glycemic disturbance profiles was generated from historical data; then, the disturbances were clustered to fit the profile to which this belongs.

#### 2.3.3 Classical control

**Figure 8.**
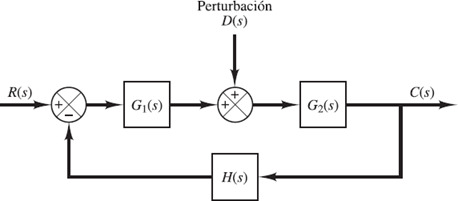
Typical feedback control scheme [43].

Classical control is a frequency domain based control approach where the process (also called system) is modeled by frequency domain equations (transfer functions) and describes how the process variable (to be controlled) changes due to known exogenous variables (inputs) or unknown ones (disturbances) [43]. The process variable is fed back and compared with a desired signal (reference) giving an error signal. Finally, the error is given to another transfer function that works like a controller that tries to minimize the error by generating an appropiated signal to the system [Figure 8].

**Figure 9.**
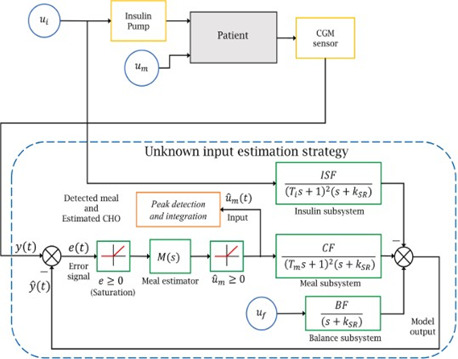
Block diagram of algorithm proposed [44].

Inspired by this approach, the carbohydrate amount (unknown input) is estimated based on a feedback scheme where measured blood glucose (BG) and a predicted BG are compared. Glycemic behavior is predicted using a personalized model considering some parameters like insulin sensitivity factor (ISF), basal insulin rate (BIR), and insulin on board (IOB) [44] [Figure 9].

The model consists of 3 transfer functions: insulin, meal, and a ficticious balance subsystem. The method has 3 stages. First, the BG prediction is based on exogenous insulin and an estimated meal. Second, is the estimation system, where an error signal is generated from read and predicted BG, which is fed to a meal estimator (transfer function). Third, the estimated meal is conditioned to be visualized in a suitable manner.

#### 2.3.4 Modern control

Modern control is a time domain based control approach where process is modeled by time domain equations (differential equations). This approach allows one to describe not only the behavior of the output of the system, but also all the internal variables (states) opening it to new, interesting, and complex control techniques.

**Figure 10.**
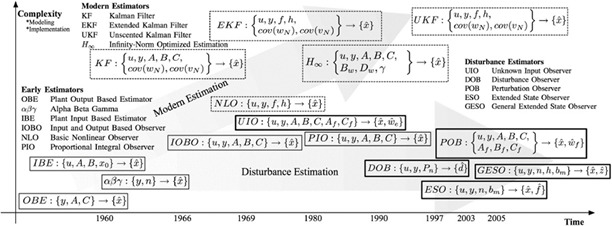
Timeline of observers [46].

Most of the proposals in this field are related to meal detection and counting turns around states observers. This basically allows masking of disturbances into dynamics that can be modeled for differential equations. Most common states observed[46] include Luenberger, proportional integral, Kalman, and so on [Figure 10].

Following this approach, we can find the next related papers. Unscented Kalman filter (UKF) is used for prediction based on the Bergman minimum model[48] and estimate personalized parameters. Meal detection is determined by the predicted value of meal interference and the difference between the predicted blood glucose concentration of UKF and the actual blood glucose concentration [47].

## 3 Commonly used datasets

In this section, we present a summary of the most commonly used datasets.

### 3.1 UVA/Padova T1DM

The UVA/PADOVA model consists of a set of differential equations, both linear and nonlinear, where each of these describes the dynamic behavior of the principal variables involved in the glucose-insulin profile in patients with type I diabetes [54]. Similarly, the simulator associated with this model, the Type I Diabetes Metabolic Simulator (T1DMS) from UVA/PADOVA, delivers the simulation of a population of 10 adults, 10 adolescents and 10 children. These T1DM populations have been generated by random extraction from distributions of joint parameters, of different realizations of the vector of parameters, that is, the vector that includes the entire set of model parameters.

### 3.2 WP7

The study will be conducted in crossover trial, with 2, 12-week periods separated by a washout period of at least one month. According to randomization, patients will be provided with either the Diabeloop system or the usual system. Patients will be trained for the use of a blood glucose meter, an external insulin pump and Diabeloop system. In both treatment periods, the same blood glucose meter will be used throughout the duration of the study. In 2 centers (Centre Hospitalier Sud-Francilien and Grenoble), a pilot study will be performed during 4 weeks to improve the efficacy of Diabeloop system with data collection, to test the manual settings by healthcare providers and patients and to check the proper functioning of the follow-up platform [55].

### 3.3 OhioT1DM

The OhioT1DM Dataset was first released in 2018 for the first Blood Glucose Level Prediction (BGLP) Challenge. At that time, the dataset was half its current size, containing data for only 6 people with type 1 diabetes. Data for an additional 6 people are being released in 2020 for the second BGLP Challenge [56].

The OhioT1DM Dataset contains 8 weeks’ worth of data for each of 12 people with type 1 diabetes. These de-identified people are referred to by randomly selected ID numbers. All data contributors were on insulin pump therapy with continuous glucose monitoring(CGM). They wore Medtronic 530G or 630G insulin pumps and used Medtronic Enlite CGM sensors throughout the 8-week data collection period. They reported life-event data via a custom smartphone app and provided physiological data from a fitness band. The first cohort of 6 individuals wore Basis Peak fitness bands. Data for this cohort were released in 2018. The second cohort of 6 individuals wore the Empatica Embrace

### 3.4 Pima Dataset

This dataset is originally from the National Institute of Diabetes and Digestive and Kidney Diseases. The objective of the dataset is to predict whether or not a patient has diabetes, based on certain diagnostic measures included in the dataset. Several constraints were placed on the selection of these instances from a larger database. In particular, all patients here are women at least 21 years old, and of Pima Indian heritage.

## 4 Results and discussion

Wide-ranging approaches and proposals have been described. Early detection and estimation techniques are common decision rule-based rather than based on more sophisti-cated procedures. Decision rules are also more feasible for easy implementation because this manages a simple logic and lower computational effort than modern approaches.

Meal detection also often faces a time-delay challenge because almost all approaches compare deviations between a prediction model and the real glucose measured, and require considerable time. In fact, it usually takes between 30 minutes and one hour later to detect the meal.

Another important problem to be resolved is the increasing number of false positive detections. In this case, there could be issues related to the quality of pre-processing of features. In fact, a good pre-processing could prevent noisy data like derivatives or even “noisy” glucose. Furthermore, It could be solved by transforming data with smoothing filters Likewise, sometimes it is possible to face an imbalanced data problem. A data imbalance refers to a classification learning problem where at least, one of the classes includes a larger number of observations than in other classes. An example of imbalanced data in diabetes could be the labels for a binary classification problem where one class represents the likelihood of a meal detected and the other the likelihood of not being detected. As is expected, the probability of having meal is lower than not having a meal because patient will not be eating all the day. Fortunately, this also could be fixed by the technique known as data augmentation. In brief, this consists of increasing the amount of data by adding modified versions of the data without affect the quality of original data.

sMoreover, newer approaches (e.g., IA-inspired) often get more accurate results, es- pecially ensemble methods. Their complexity and depth offers insights that not could be reached from others techniques. That is the case of recurrent neural networks like LSTM, GRU, and BILSTM which learn long-term dependencies between time steps of sequential data. The majority of proposals are performed on in silico patients rather than in vivo ones. One of most important and reported for trials in in silico databases is the T1DM UVA/PADOVA simulator. This is the first (and currently only) in silico diabetes model accepted by the Food and Drug Administration as a substitute for pre-clinical animal testing of new treatment strategies for type 1 diabetes mellitus. Additionally, this allows one to simulate not only glucose, but also meal profiles, insulin treatment, time of simulation, and control algorithms.

Despite its popularity, UVA/PADOVA, and in general, synthetic data could not be a good idea for trials. In fact, in silico records are often non-realistic data because they do not cover other important features like stress, illness, or sleep quality, for instance. Real data are noisy, sometimes, lacking information like meal announcements, or glucose recordings due to faults in the monitoring system. Real data also are nonlinear, the same meal eaten in different times of the day could not produce exactly the same effect on glucose.

Most procedures require as inputs glucose samples from continuous glucose monitoring devices such as basal insulin and bolus. Most CGM samples glucose require 5 minutes. Most approaches could be grouped by 2 categories: mathematical model based and not model based. Meal detection times are usually delayed yielding delayed meal estimation too. Glucose dynamics often changes not only due to insulin and meals, but also due to stress, physical exercise, or sleeping hours which are not usually taken into account by algorithms.

## Abbreviations

DM: Diabetes mellitus

T1DM: Type 1 Diabetes Mellitus

IDF: International Diabetes Federation

IIT: Intensive Insulin Therapy

MDIs: Multiple Daily Injections

CSII: Continuous Subcutaneous Insulin Infusion

AP: Artificial Pancreas

CGM: Continuous Glucose Monitoring

MHE: Moving Horizon Estimation

AUC: Are Under Curve

ROC: Rate of Change

RNNs: Recurrent Neural Networks

LSTM: Long Short Term Memory

SEQ2SEQ Sequence to Sequence

RL: Reinforcement Learning

DOBC: Disturbance Observer Based Control

SMC: Sliding Mode Control

SMO: Sliding Mode Observer

RA: Rate of Aparison

FOSMO: First Order Sliding Mode Observer

MPC: Model Predictive Control

BG : Blood Glucose

ISF: Insulin Sensitivity Factor

BIR: Basal Insulin Rate

IOB: Insulin on Board

UKF: Unscented Kalman Filter

BGLP: Blood Glucose Level Prediction
